# Active form of AKT controls cell proliferation and response to apoptosis in hepatocellular carcinoma

**DOI:** 10.3892/or.2013.2932

**Published:** 2013-12-16

**Authors:** IMGE KUNTER, ESRA ERDAL, DENIZ NART, FUNDA YILMAZ, SEDAT KARADEMIR, OZGUL SAGOL, NESE ATABEY

**Affiliations:** 1 Department of Medical Biology and Genetics, Faculty of Medicine, Dokuz Eylul University, Inciralti, Izmir 35340, Turkey; 2 Department of Pathology, Faculty of Medicine, Ege University, Bornova, Izmir 35100, Turkey; 3 Department of General Surgery, Faculty of Medicine, Dokuz Eylul University, Inciralti, Izmir 35340, Turkey; 4 Department of Pathology, Faculty of Medicine, Dokuz Eylul University, Inciralti, Izmir 35340, Turkey

**Keywords:** AKT, hepatocellular carcinoma, proliferation

## Abstract

Hepatocellular carcinoma (HCC) is the third most common cause of cancer-related mortality worldwide. Deregulation of the AKT signaling pathway has been found in HCC. However, the effect of AKT activation on the proliferation and apoptosis in HCC is not clear. Herein, expression of phosphorylated form of AKT (Ser 473) was investigated in HCC tumor (n=73), cirrhosis (n=17), normal liver (n=22) samples and in HCC cell lines (n=8). The results showed that expression of p-AKT was higher in tumor (53%) than in cirrhotic tissues (12%) while it was absent in normal liver (p<0.0001). p-AKT expression was also associated with number of tumor nodules and differentiation status (p<0.05). LY294002 induced cell cycle arrest at G0/G1 in SNU-449 and Mahlavu cells by decreasing expression of CDK2, CDK4, CycD1, CycD3, CycE, CycA and increasing expression of p21 and p27 as well; it also caused a decrease in the E2F1 transcriptional activity through declining phosphorylated Rb. LY294002 did not affect the basal level of apoptosis; however, it amplified cisplatin-induced apoptosis in SNU-449 cells. When the p-AKT level was decreased specifically after transfection with the DN-AKT plasmid, SNU-449 cells became more sensitive to cisplatin-induced apoptosis. HuH-7 cells with no basal p-AKT, were markedly affected by the treatment of doxorubicin. Thus, Akt signaling controls growth and chemical-induced apoptosis in HCC and p-AKT may be a potential target for therapeutic interventions in HCC patients.

## Introduction

Hepatocellular carcinoma (HCC) is the fifth most common cancer worldwide and a major form of liver cancer responsible for 90% of the primary malignant liver tumors in adults (1). Most patients have a low survival rate due to locally advanced or metastatic diseases and surgery is feasible for only a low percentage of patients with HCC. Although chemotherapy is ineffective due to toxicity and poor response, recent trials with appropriate patient selection and targeted molecular therapeutics are promising in the treatment for advanced HCC (2,3). Sorafenib, a multiple tyrosine kinase inhibitor, is one of the promising drugs clinically approved for patients with advanced HCC. However, the response rate to sorafenib in HCC is very low (2–3%) and it has been shown that activation of the phosphoinositide 3-kinase/AKT (PI3K/AKT) signaling pathway mediates acquired resistance to sorafenib in HCC (4). Therefore, development of novel strategies for HCC treatment is required.

PI3K/AKT signaling is one of the major signaling pathways activated in human cancer including HCC and is therefore considered a suitable molecular target (5). Several PI3K inhibitors are in the clinic and many more are in preclinical development. It has been shown that novel inhibitors targeting the PI3K signaling cascade cause decrease in cell proliferation and in some circumstances promote cell death of HCC cells *in vitro* (6–8). There are contradictory results regarding the effect of PI3K inhibition on apoptosis and cell cycle in different cancer types including HCC. Two PI3K inhibitors, LY294002 and ZSTK474, were found to suppress cell growth without inducing apoptosis (9). Dan *et al* were demonstrated that the inhibition of AKT suppressed proliferation by decreasing expression of CycD1 and Ki-67, while not increasing apoptotic cell numbers in six different cell lines from four different cancer models and human cancer xenografts (9). In contrast, another study showed that LY294002 induces apoptosis of human nasopharyngeal carcinoma *in vitro* and *in vivo* (10). Moreover, it has been reported that PI3K-mTOR inhibition does not promote substantial apoptosis in the EGFR mutant lung cancer while it induced apoptosis in HER2-amplified breast cancer (11). In EGFR mutant or KRAS mutant lung cancer models, tumor regression associated with apoptosis was also observed only when the PI3K/AKT pathway and MEK/MAPK pathway were simultaneously blocked (12). Thus, the literature suggests that the effect of inhibition of PI3K signaling might cause different effects in a context-dependent manner. Little is known about the effect of PI3K/AKT inhibition on the cell cycle and apoptosis in HCC.

In the present study, we first analyzed the activation status of AKT in normal liver, cirrhotic, HCC tissues and HCC cell lines. Then, we functionally analyzed the effect of AKT inhibition on cell proliferation and apoptosis by explaining how the level of activated form of AKT induces apoptosis in HCC cell lines.

## Materials and methods

### Cell culture

Human HCC cell lines (Mahlavu, SNU-449, SNU-475, HepG2, PLC/PRF/5, SNU-398, HuH-7, Hep3B) were provided by Dr Mehmet Öztürk (Bilkent University, Turkey). Cells were maintained in DMEM with 10% FBS, 100 U/ml penicillin, 2 mM L-glutamine, and 100 mg/ml streptomycin in 5% CO_2_ at 37°C (Biological Industries, Israel). LY294002 (Calbiochem, Nottingham, UK) was used to inhibit AKT signaling pathway, doxorubicin and cisplatin were used as an apoptotic inducer.

### Western blotting

Western blotting was performed as previously described (13). For immunoblotting p-AKT Ser 473(CS-4051), AKT (CS-7292), p-Rb Ser 608 (CS-2181), p-Rb Ser 780 (CS-9307), p-Rb Ser 795 (CS-9301), p-Rb Ser 807/811 (CS-9308), Rb (CS-9309), p-MAPK p44/p42 (ERK1/2) Thr202 Tyr204 (CS-4377), p21/Cip1/waf1(CS-2946), p27 (sc-1641), p18 (sc-9965), CycE (sc-247), CycA (sc-239), CycD1 (sc-718), CycH (sc-855), CycD3 (sc-6283), CDK2 (sc-6248), CDK4 (sc-601), CDK6 (sc-177) and CDK7 (sc-7344) and Calnexin (sc-11397) antibodies were used. Detection was performed by Super Signal West Dura Extended Duration Substrate (Pierce, IL, USA).

### Cell proliferation analyses with BrdU incorporation

DNA synthesis in LY294002-treated and -untreated cells was determined by BrdU incorporation. Cells were seeded at a density of 20×10^3^ cells/well in 12-well plates. BrdU (30 μM) (Darmstadt, Germany) was added to media 4 h before ethanol fixation. Following DNA denaturation, cells were incubated with anti-BrdU monoclonal antibody (Dako, Denmark). Peroxidase labeled IgG was used as secondary antibody and 3,3′-diaminobenzidine tetrahydrochloride (DAB) substrate (Dako) was also used for visualization. Cells were counterstained by hematoxylin. Positively stained cells were counted with a light microscope and the cell growth ratio (%) was calculated by dividing the number of BrdU positive nuclei by the total number of nuclei.

### Cell cycle analysis

Cell cycle distribution was quantified by flow cytometry. Cells were trypsinized at 24 and 48 h after treatment with LY294002. Pellets were resuspended and fixed in ethanol. After washing, cells were incubated in 0.1% Triton X-100 and DNAse-free RNAse (200 mg/ml), then stained with propidium iodide. Cells were analyzed by BD FACSCanto version 5.03 Flow Cytometry and Cell cycle distribution was analyzed by using BD FACS Diva version 5.03. and Modfit LT 3.0 software (BD Biosciences, FACSCanto, San Jose, CA, USA).

### Luciferase reporter assays

Cells were transiently transfected with both the E2F1 luciferase reporter construct [pGL33 + TAP73 (−833) E2F1 responsive vector, kindly provided by Dr Emre Sayan] and the pRL-TK plasmid vector (kindly provided by Mehmet Ozturk) by using FuGENE HD transfection reagent (Roche, Mannheim, Germany). Cells were harvested and luciferase activity was measured using the Dual Luciferase Reporter Assay kit (Promega, Madison, WI, USA) according to the manufacturer’s instructions. Levels of reporter gene induction in transfected cells were calculated by normalizing the Firefly luciferase levels to total Renilla luciferase levels.

### Detection of apoptosis

The Annexin V-FITC Apoptosis Detection kit (BD Biosciences) was used to analyze apoptosis by flow cytometry according to the manufacturer’s recommendations. Cells were transiently transfected with pBabe puroL Akt K179M T308A S473A (ΔN-AKT) plasmid (kindly provided by Aykut Uren) for 48 h. They were then treated with 10 μM cisplatin, 5 and 10 μM doxorubicin and/or 25 μM LY294002 for both 24 and 48 h before staining with Annexin V. Cells were analyzed by flow cytometry (FACSCalibur; BD Biosciences) and results were analyzed by BD CellQuestPro.

### Immunohistochemistry

Formalin-fixed, paraffin-embedded liver tissue samples were obtained from 73 patients with HCC in cirrhotic background and 17 patients with cirrhosis who underwent complete resection without any preoperative treatment in Dokuz Eylül and Ege University Hospitals in Izmir, Turkey. Normal liver biopsies were used as controls (n=22). The study was approved by the Ethics Committees of both Dokuz Eylül and Ege University Medical Schools. Written informed consent was obtained from all patients prior to liver transplantation or liver biopsy sampling. Tissue sections were stained with hematoxylin and eosin (H&E) and then examined by light microscopy. The histopathological diagnosis of all patients was carried out according to the WHO histopathological classification of liver disease. Immunostaining was performed using an automated immunohistochemical stainer according to the manufacturer’s guidelines (Biogen, Lab Vision Autostainer 360, CA, USA). The antigen retrieval step was performed with proteinase K. Endogenous peroxidase and avidin activities were blocked by 3% H_2_O_2_ and the avidin-biotin block solution, respectively. Sections were then incubated with anti-p-AKT antibody (CS-4051) at 1:100 dilutions. The reaction product was developed using 3,3′-diaminobenzidine tetrahydrochloride (DAB) and sections were counterstained with hematoxylin. Sections were then evaluated by two pathologists (O.S., F.Y., D.N.). The extent of p-AKT expression was semi quantitatively graded as 0 (no stain), 1+ (<20% of the cells), 2+ (positivity between 20–60% of cells), and 3+ (>60% of cells).

### Statistical analysis

All graphic data are expressed as the mean ± SE. Statistical analysis of data was performed using the GraphPad Prism and Statistical Package for Social Sciences 15.0 (SPSS Inc., Chicago, IL, USA). Analysis of variance (ANOVA) was used to determine the significance of the differences between means of multiple groups. The association of p-AKT expression of tissue samples and clinicopathological characteristics of patients was examined with the χ^2^ test. p<0.05 was considered to indicate a statistically significant difference.

## Results

### p-AKT expression markedly decreases from HCC tissues to normal liver tissues

**Figure 1:**
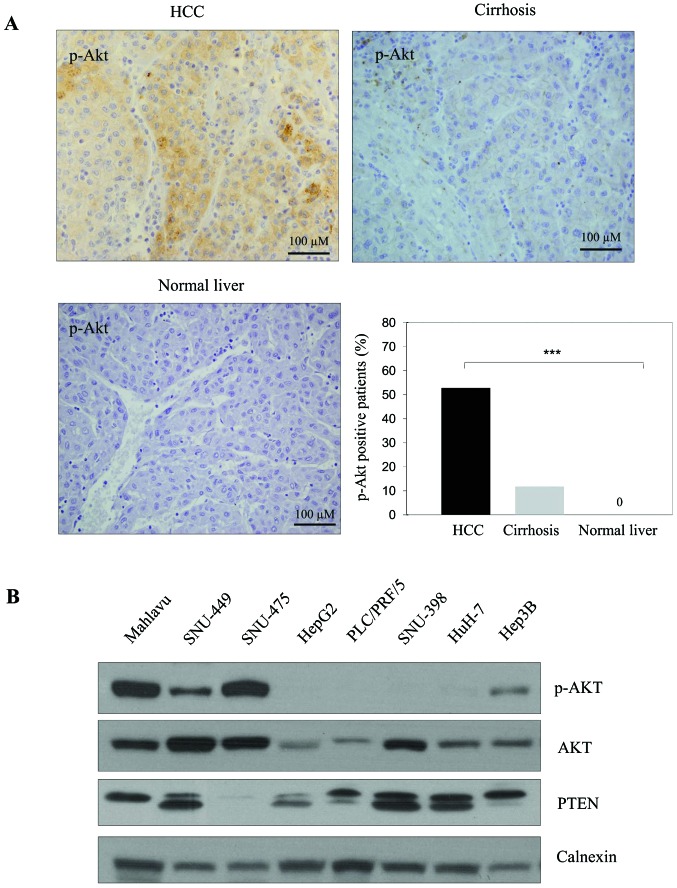
Expression of active phosphorylated form of AKT (ser 473) in HCC. (A) Immunohistochemical analysis of p-AKT expression in HCC (n=73), cirrhotic (n=17) and normal liver (n=22) tissue sections (^***^p<0.0001). (B) Western blot analysis for the expression of p-AKT, total AKT and PTEN in HCC cell lines (n=8). Calnexin was performed as an equal loading control. These results are representative of 3 individual experiments.

The p-AKT expression was observed in 53% of HCC tissues and in 12% of cirrhotic tissues (Fig. 1A). Although the p-AKT staining was significantly strong in HCC tumor tissues, little or no staining was observed in cirrhotic and normal liver tissues, respectively (p<0.001). p-AKT signaling was higher in HCC patients with more than three nodules than in patients with less than three. Furthermore p-AKT level was significantly higher in patients with moderate/poorly differentiated HCC than in patients with well differentiated tumors (Table I). There was no significant correlation between activated AKT level and tumor size or gender.

### PI3K/AKT signaling is often active in HCC cell lines

The p-AKT expression was high in Mahlavu and SNU-475, moderate in Hep3B and SNU-449 and almost absent in HepG2, PLC/PRF/5, SNU-398 and HuH-7 cells. The expression of total AKT was observed at different levels in these cell lines. When PTEN level was tested as a negative upstream regulator of PI3K/AKT signaling, a positive correlation was found between dephosphorylated functional PTEN and p-AKT levels (Fig. 1B). This suggests functional PTEN might block Akt phosphorylation as expected.

### LY294002 treatment inhibits AKT activation in a dose-dependent manner in both Mahlavu and SNU-449 cell lines

**Figure 2:**
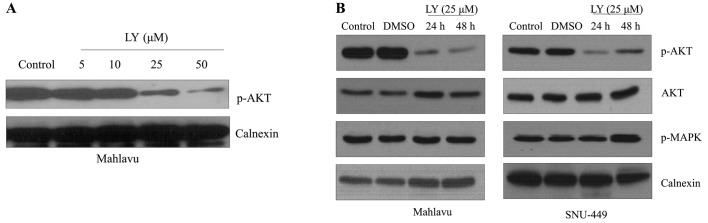
Dose- and time-dependent inhibition of PI3K/AKT signaling by LY294002. (A) Dose-dependent inhibition of p-AKT expression in the Mahlavu cells. (B) Time-dependent effect of LY294002 on the expression of p-AKT, total AKT and active form of MAPK as a downstream signaling molecule in both SNU-449 and Mahlavu cells. Calnexin blotting was performed as an equal loading control. These results are representative of at least three individual experiments.

To define the influence of AKT activation on the proliferation of HCC cell lines, we first analyzed the dose-dependent effect of LY294002 on the active phosphorylated form of AKT in Mahlavu cells. LY294002 treatment for 48 h resulted in a progressive decrease in the p-AKT level in Mahlavu cells in a dose-dependent manner (Fig. 2A). Since the MAPK signaling pathway is one of the best-characterized signaling pathways that regulate cell proliferation and there is a cross-talk between Ras/MAPK and PI3K/AKT signaling at different levels in a context-dependent manner (14), we further analyzed the effect of LY294002 on the expression of Phospho-p44/42 MAPK (Erk1/2) (Thr202/Tyr204). The inhibition of AKT signaling did not affect activation of MAPK signaling in HCC cell lines (Fig. 2B).

### Inhibition of AKT induces cell cycle arrest at G0/G1 and increases sensitivity to chemical induced-apoptosis in HCC cell lines

**Figure 3:**
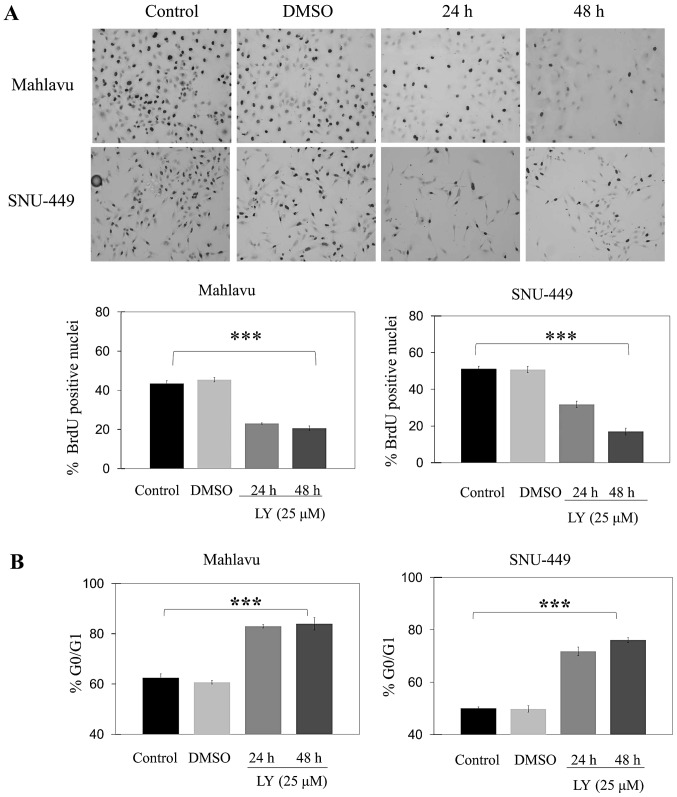
Effect of PI3K/AKT inhibition on the proliferation of HCC cell lines. (A) The BrdU incorporation assay was used to determine number of dividing cells under the time-dependent effect of LY294002 on SNU-449 and Mahlavu cells. BrdU positive cells were quantified as percentage of >2,000 cells (^***^p<0.0001). (B) Percentage of G0/G1 cells in Mahlavu and SNU-449 cells after LY294002 treatment (^***^p<0.0001). Data include mean ± SE values from three independent experiments and each experiment was performed in triplicate.

**Figure 4:**
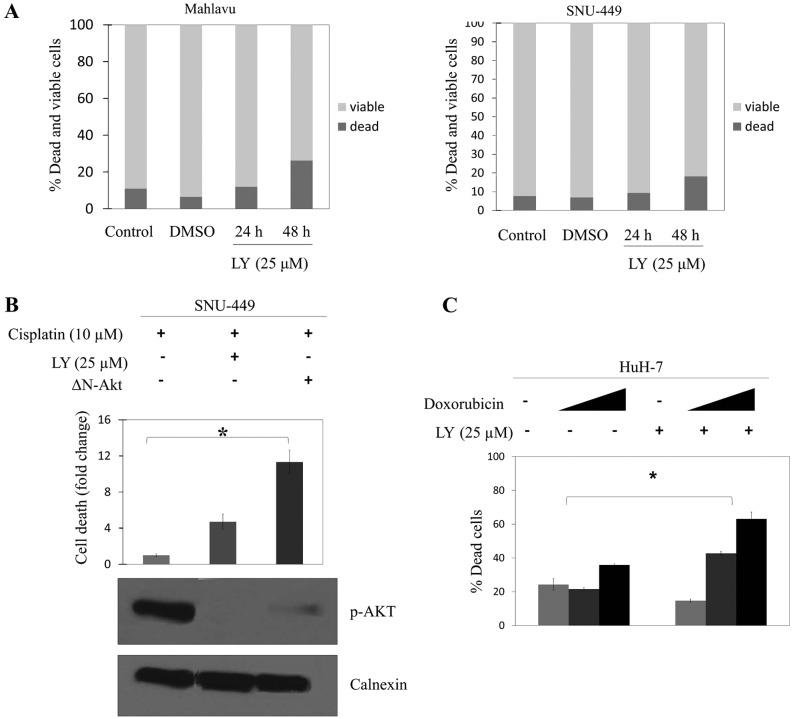
Effect of the PI3K/AKT inhibition on the basal and chemical-induced apoptosis in HCC cell lines. (A) Time-dependent effect of LY294002 on the apoptosis in Mahlavu and SNU-449 cells. (B) Effect of PI3K/AKT inhibition either by LY294002 treatment or the transient transfection of ΔN-Akt on the cisplatin-induced apoptosis in SNU-449 cells (^*^p<0.05). Data include mean ± SE values from three independent experiments. (C) Effect of LY294002 on the doxorubicin-induced (5–10 μM) apoptosis in HuH-7 cells.

Treatment with LY294002 caused a significant decrease in the percentage of cells in S phase at 24 and 48 h as compared to controls in both Mahlavu and SNU-449 cell lines (Fig. 3A). LY294002 caused a significant (p<0.001) increase in the percentage of cells at G0/G1 phase at 24 and 48 h in both Mahlavu and SNU-449 (Fig. 3B). Furthermore, there was no statistically significant difference in the apoptotic death in either Mahlavu or SNU-449 cells after treatment of LY294002 for 24 h (Fig. 4A). However, 48 h LY294002 treatment amplified cisplatin-induced apoptosis in SNU-449 cells. When the p-AKT level was decreased specifically by transient transfection of ΔN-AKT plasmid into SNU-449 cells, they became even more sensitive to apoptosis induced by cisplatin (Fig. 4B). HuH-7 cells which do not have basal phosphorylated AKT were markedly affected and killed by the treatment of doxorubicin in a concentration-dependent manner (Fig. 4C).

### LY294002-induced G0/G1 arrest is correlated with differential expression of cell cycle effectors

**Figure 5:**
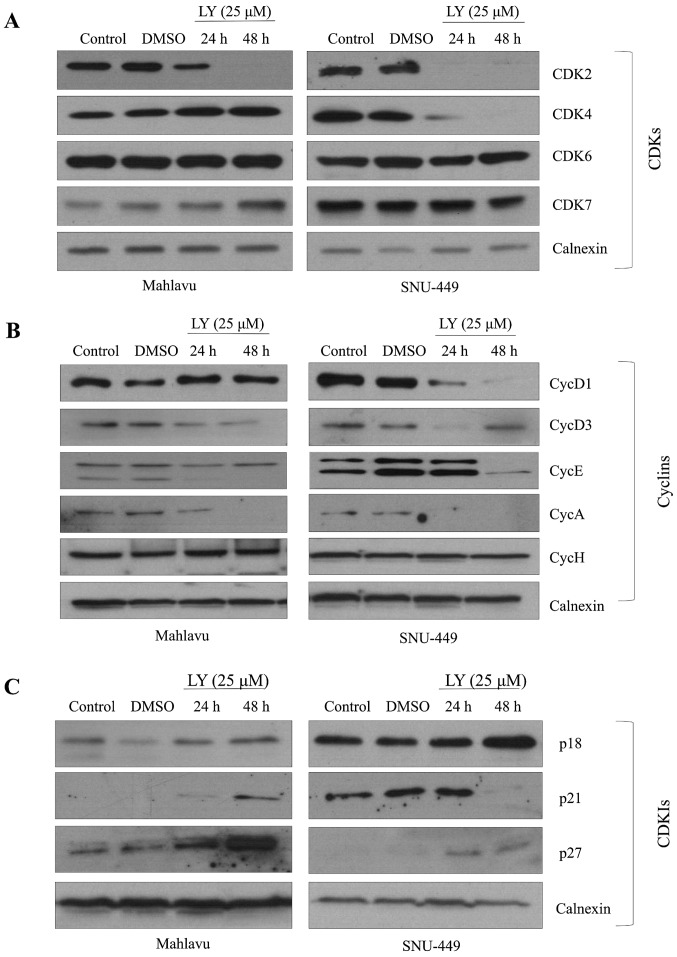
Effect of the inhibition of PI3K/AKT signaling on the expression of cell cycle regulator genes in HCC cell lines. (A) Differential expression of cyclin-dependent kinases (CDK2, CDK4, CDK6 and CDK7), (B) cyclins (CycD1, CycD3, CycE, CycH and CycA), (C) cyclin-dependent kinase inhibitors (p18, p21 and p27) in the time-dependent effect of LY294002 in Mahlavu and SNU-449 cells. Calnexin blotting was performed as an equal loading control. These results are representative of at least three individual experiments.

To understand the mechanisms behind G1 arrest under the effect of PI3K/AKT inhibition, G1 cell cycle arrest-related molecules were analyzed. We showed that LY294002 decreased CDK2 expression strongly while it did not affect the expression of CDK6 in both Mahlavu and SNU-449 cells. Meanwhile, inhibition of AKT caused a decrease in the expression of CDK4 in the SNU-449 cell line, but not in Mahlavu cells (Fig. 5A). LY294002 reduced expression of CycD1 and CycE in both SNU-449 and Mahlavu. Since CycD3, an isoform of CycD, plays an important role especially in the proliferation of liver cells, we further analyzed expression of CycD3 after PI3K inhibition. It caused a significant decrease in the expression of CycD3 in both HCC cell lines. Moreover, we found that there was no expression of CycA as S phase regulator under the PI3K/AKT inhibition in both cell lines. CycH functions as a CDK activating kinase by forming complex with CDK7. Both CycH and its kinase partner participate in a transcriptional regulation process during cell cycle. PI3K/AKT inhibition did not affect CycH and CDK7 expression levels in SNU-449 and Mahlavu cells (Fig. 5B). We also analyzed the expression of CDKIs and observed that LY294002 treatment increased the levels of p27 and p21 expression in both cell lines, as it caused a moderate increase in the level of p18 expression in the SNU-449 cell line (Fig. 5C).

**Figure 6:**
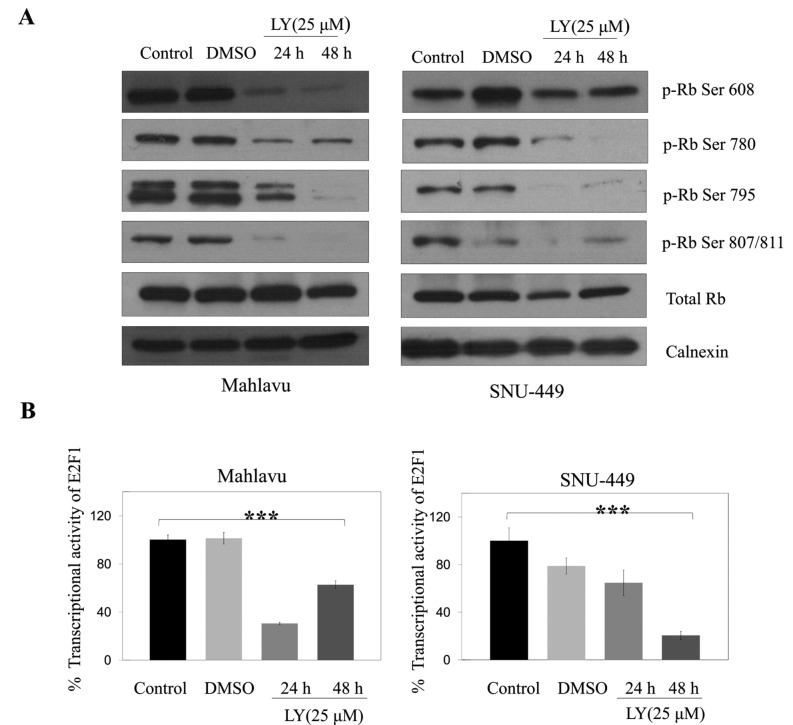
Decrease in the Rb/E2F1 activity through the inhibition of PI3K/AKT signaling in HCC cell lines. (A) Expression of total Rb and hyperphosphorylated forms of Rb (Ser 608, 780, 795, 807/811). (B) Transcriptional activity of E2F1 after time-dependent LY294002 treatment in Mahlavu and SNU-449 cells (^***^p<0.0001). Data include mean ± SE values from three independent experiments and each experiment was performed in triplicate.

Retinoblastoma protein (Rb) is inactivated by CDK-dependent phosphorylation or by proteolytic degradation, and downregulation of CDK4/6 has also been associated with low level of Rb (15). Since LY294002 reduced the amount and potential activity of CDK4 and CDK6 in SNU-449 and Mahlavu cells, we examined the levels and phosphorylation status of Rb in these cells. LY294002 treatment strongly decreased levels of different phosphorylated forms of Rb (Ser 608, Ser 780, Ser 795, Ser 807/811) and no difference in the level of total Rb was observed in either Mahlavu or SNU-449 cell lines (Fig. 6A) consistent with the inhibition of cell cycle arrest. When we examined transcriptional activity of E2F1 controlled by Rb phosphorylation, consistent with the decrease in hyperphosphorylated form of Rb, inhibitor treatment caused a significant (p<0.001) decrease in the level of E2F1 activity over 24 and 48 h in Mahlavu and only over 48 h in SNU-449 cells (Fig. 6B).

## Discussion

The PI3K/AKT signaling pathway plays a pivotal role in hepatocarcinogenesis; therefore, it is an attractive candidate as an anticancer drug target for HCC treatment (2,3,5,16). Aberrant activation of the PI3K/AKT pathway is often found in different types of human cancer, including HCC, which might be explained by the hotspot PI3KCA mutations and AKT gene amplifications as well as the increase in phosphorylation of AKT (17).

In the present study, we analyzed the activated form of AKT (p-Ser 473) to define the activation status of PI3K/AKT signaling pathway in HCC patients. Immunostaining showed a very high degree of positive staining for the expression of p-Ser 473 AKT as 38 of 73 (53%) among HCC tissues describing high activation level of PI3K/AKT signaling pathway. There was also little and/or no staining for the activated form of AKT in cirrhotic and normal liver tissues. Consistent with our data, Li *et al* (18) reported that immunoreactivity score of the activated AKT was very low (0–3) in >80% of cirrhotic and normal tissues, while it was high (4–6) in 65% of HCC patient tissues. Furthermore, Schmitz *et al* (19) found that there was no staining for p-AKT in normal liver and increased p-AKT expression was significantly associated with a lower, overall disease-specific survival. As a result, our data, in agreement with other studies, suggested that the p-AKT level can be used as a prognostic and early disease recurrence risk factor in different studies based on HCC patient data (18–20).

In accordance with our immunohistochemistry data, increased levels of activated AKT were also observed in 5 of the 8 (62.5%) HCC cell lines that we tested and were correlated with the level of PTEN expression. Moreover, p-AKT expression was positively correlated with the number of nodules in HCC patients and was also significantly higher in poorly differentiated HCC patient samples when compared with well-differentiated ones.

To test the effect of AKT inhibition on the cell cycle progression of HCC cells, we then performed cell cycle analyses by flow cytometry and BrdU incorporation assay. Results clearly indicated that G1 arrest occurred and the number of cells entering S phase significantly decreased after LY294002 treatment. G1 arrest in these cells was correlated with the downregulation of the expression of CycD1, CycD3, CycE, CycA, CDK2 and CDK4 as well as decreased retinoblastoma protein activity. However, the inhibition of AKT by LY294002 did not induce basal apoptotic machinery in either the SNU-449 or the Mahlavu cells. There are contradictory results on the effect of AKT inhibition on apoptosis in different human cancer types. For instance, the PI3K inhibitor LY294002 triggers apoptosis in colorectal cancer (21), pancreatic cancer (22) and androgen-sensitive prostate cancer (23). However, Dan *et al* (9), demonstrated that both PI3K inhibitors, LY294002 and ZSTK474, suppress proliferation by decreasing expression of CycD1 and Ki-67, while they do not increase apoptosis in prostate cancer, lung cancer, glioblastoma and colorectal cancer cell lines and in human cancer xenografts. Additionally, Faber *et al* (11), showed that the PI3K-mTOR inhibition does not promote substantial apoptosis in EGFR mutant lung cancer while it induces apoptosis in HER2-amplified breast cancer.

Moreover, in EGFR mutant or KRAS mutant lung cancer models, tumor regression associated with apoptosis was observed only when the PI3K/AKT pathway and MEK/MAPK pathway were simultaneously blocked (9,24). In our study, the inhibition of the PI3K/AKT pathway by LY294002 treatment did not affect the MEK/MAPK pathway and caused growth inhibition without apoptosis. When we decreased the level of phosphorylated form of AKT by transient transfection of ΔN-AKT and treated with LY294002 in SNU-449 cells, the level of apoptosis was increased independent of cisplatin treatment. In light of these results, the level of activated AKT may regulate apoptotic response in basal or induced conditions in HCC cells. Collectively, our data suggest that overexpression of the activated AKT may be a biomarker for the prognosis of HCC. The increased p-AKT level may, on the other hand, be a biomarker for predicting treatment response when conventional chemotherapeutic agents are used as apoptotic inducers.

## Figures and Tables

**Table I:** Clinicopathological characteristics of primary hepatocellular carcinoma patients.

Characteristics	No. of patients	p-AKT		P-value

		(+)	(%)	
Gender				
Female	10	5	50	1.000
Male	43	24	55	
Tumor size				
<5 cm	32	15	46	0.360
≥5 cm	10	7	70	
No. of tumor nodules				
<3	22	8	36	0.038^a^
≥3	23	16	70	
Tumor grade				
Well differentiated	9	3	33	0.01^a^
Moderately differentiated	27	15	55	
Poorly differentiated	7	4	57	

a P-values were calculated using Chi-square analysis.
